# Obesity and Clonal Hematopoiesis of Indeterminate Potential: Allies in Cardiovascular Diseases and Malignancies

**DOI:** 10.3390/life13061365

**Published:** 2023-06-10

**Authors:** Luka Komic, Marko Kumric, Hrvoje Urlic, Azer Rizikalo, Marko Grahovac, Jelena Kelam, Marion Tomicic, Doris Rusic, Tina Ticinovic Kurir, Josko Bozic

**Affiliations:** 1 Department of Family Medicine, Split-Dalmatia County Health Center, 21000 Split, Croatia; luka.komic@dz-sdz.hr (L.K.); jelena.kelam@dz-sdz.hr (J.K.); marion.tomicic@mefst.hr (M.T.); 2 Department of Pathophysiology, University of Split School of Medicine, 21000 Split, Croatia; marko.kumric@mefst.hr (M.K.); hrvoje.urlic@dz-sdz.hr (H.U.); tticinov@mefst.hr (T.T.K.); 3 Laboratory for Cardiometabolic Research, University of Split School of Medicine, 21000 Split, Croatia; 4 Department of Anatomy, School of Medicine, University of Mostar, 88000 Mostar, Bosnia and Herzegovina; azer.rizikalo@mef.sum.ba; 5 Department of Pharmacology, University of Split School of Medicine, 21000 Split, Croatia; marko.grahovac@mefst.hr; 6 Department of Family Medicine, University of Split School of Medicine, 21000 Split, Croatia; 7 Department of Pharmacy, University of Split School of Medicine, 21000 Split, Croatia; doris.rusic@mefst.hr; 8 Department of Endocrinology, Diabetes and Metabolic Diseases, University Hospital of Split, 21000 Split, Croatia

**Keywords:** obesity, clonal hematopoiesis of indeterminate potential, CHIP, cardiovascular disease, malignancy, leukemia

## Abstract

The clonal hematopoiesis of indeterminate potential (CHIP) is a term used to describe individuals who have detectable somatic mutations in genes commonly found in individuals with hematologic cancers but without any apparent evidence of such conditions. The mortality rate in individuals with CHIP is remarkably higher than the influence ascribed to hematologic malignancies, and it is plausible that cardiovascular diseases (CVD) could elucidate the apparent disparity. Studies have shown that the most frequently altered genes in CHIP are associated with the increased incidence of CVDs, type 2 diabetes mellitus (T2DM) and myeloid malignancies, as well as obesity. Additionally, multiple research studies have confirmed that obesity is also independently associated with these conditions, particularly the development and progression of atherosclerotic CVD. Considering the shared pathogenetic mechanisms of obesity and CHIP, our objective in this review was to investigate both preclinical and clinical evidence regarding the correlation between obesity and CHIP and the resulting implications of this interaction on the pathophysiology of CVDs and malignancies. The pro-inflammatory condition induced by obesity and CHIP enhances the probability of developing both diseases and increases the likelihood of developing CVDs, T2DM and malignancies, suggesting that a dangerous vicious loop may exist. However, it is vital to conduct additional research that will suggest targeted treatment options for obese individuals with CHIP in order to reduce harmful effects connected to these conditions.

## 1. Introduction

The production of various blood cells, including erythrocytes, platelets and white blood cells, is known as hematopoiesis. The initial cell in this process is a multipotent hematopoietic stem cell (HSC), a cell that sits atop a hierarchy of progenitors in the bone marrow of adult mammals. HSC has two key abilities: to self-renew or to differentiate into a multipotent progenitor (MPP) from which all mature blood lineages are formed [1]. If the HSC generates MPP, the first division occurs between the MPP-derived common myeloid progenitors (CMP) and the common lymphoid progenitors (CLP), while the latter differentiates into T-lymphocytes, B-lymphocytes, NK and dendritic cells. Conversely, CMP separates into two types of progenitors: bipotent granulocyte-macrophage (GMP) and megakaryocyte–erythrocyte progenitor (MEP). GMP then forms granulocytes and monocytes while MEP differentiates into megakaryocytes and erythrocytes [2].

Throughout the intricate process of differentiation, it is expected that somatic mutations will occur. Typically, this involves the spontaneous transformation of 5-methylcytosine to thymine through deamination. If the error is not corrected before replication, one of the daughter cells will possess an erroneous base pair. This process advances linearly over time and is therefore viewed as a hallmark of aging [3,4]. Another type of mutation are small indels which can lead to frameshift mutations if they occur within the protein-coding segments of the genome [3,5]. Mistakes during DNA polymerase replication are yet another form of mutation [3,6]. Furthermore, there is a chance of significant changes in structure, including deletions, insertions, the loss of heterozygosity or rearrangements that cover several kilobases or more, although these types of mutations are less common than base substitutions and small indels [3,7]. As a person ages, the amount of these previously stated genetic alterations amplifies. It is projected that by the time a person reaches the age of 70, they may have up to 1.4 million protein-coding variations in their HSC reservoir. In case any of these mutations lead to a fitness advantage of the impacted cell, it will initiate clonal expansion through the process known as clonal hematopoiesis (CH). CH typically pertains to any clonal expansion of hematopoietic cells, irrespective of the underlying cause or health condition [3].

One of the CH subgroups, referred to as the clonal hematopoiesis of indeterminate potential (CHIP), is utilized to designate individuals who exhibit noticeable somatic clonal mutations in genes that are frequently mutated in hematologic cancers, yet do not exhibit any recognized hematologic malignancies or other clonal ailments [8]. However, studies have consistently demonstrated that CHIP substantially increases the risk of myeloid malignancies. Moreover, recent evidence suggests that mortality in individuals that bear such mutations is much higher, and thus could not be attributed to a transformation into acute leukemia, and that cardiovascular diseases (CVD) might account for the observed gap. In line with this, obesity, a well-established risk factor for CVD, was also associated with increased risk of leukemias.

In consideration of the overlapping pathogenetic mechanisms between obesity and CHIP, in the present review, we aimed to explore preclinical and clinical evidence concerning the association between CHIP and obesity, as well as the consequent implications of such an interaction on the pathophysiology of cancer and CVD.

## 2. The Definition of CHIP

As an increasing number of individuals undergo genetic testing for personal, research or medical purposes, the identification of CHIP among the population has also been on the rise [8]. In order to accurately determine whether an individual has CHIP, the criteria established by Steensma et al. should be satisfied [8]:

- No indication of morphological characteristics supporting any hematological malignancy.
- Elimination of paroxysmal nocturnal hemoglobinuria (PNH), monoclonal gammopathy of undetermined significance (MGUS) and monoclonal B-cell lymphocytosis (MBL) as a possible diagnosis.
- Proof of a somatic mutation linked to a hematologic neoplasm with a minimum allele frequency of 2% (e.g., *DNMT3A*, *TET2*, *ASXL1* and others).

As a group of patients that undergoes the molecular genetic testing of peripheral blood or bone marrow for diagnostic reasons will include patients with cytopenia, it is essential to determine the classification of these individuals in case any of the hematologic neoplasm-related mutations are detected [8,9]. When an individual experiences cytopenia without any indication of clonality or dysplasia, it is referred to as idiopathic cytopenia of undetermined significance (ICUS). If one or more somatic mutations are present without any dysplasia, it falls under the CHIP subgroup named the clonal cytopenias of undetermined significance (CCUS). Lastly, if there is evidence of clonality and dysplasia, the person is diagnosed with myelodysplastic syndrome (MDS) or acute myeloid leukemia (AML), depending on the percentage of blasts [8].

## 3. Somatic Mutations in CHIP

**Figure 1:**
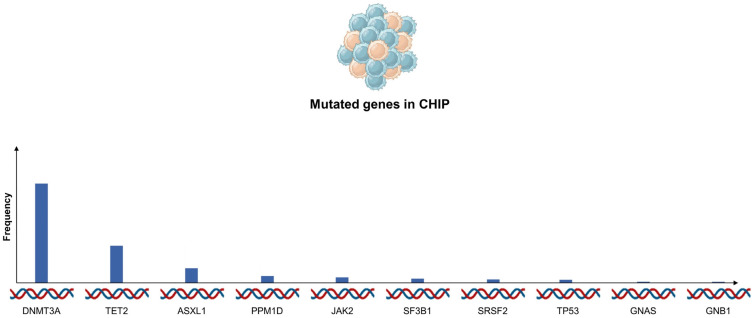
Frequency of mutated genes in CHIP (TOPMed cohort). Blue columns represent frequency of respective mutated genes. Abbreviations: CHIP—clonal hematopoiesis of indeterminate potential.

The most frequently altered genes in CHIP are *DNMT3A*, *TET2* and *ASXL1*, which encompass 75% of all CHIP cases (Figure 1) [10,11,12,13].

*DNMT3A* is the most frequently mutated gene and accounts for more than half of all CHIP cases [13]. As an epigenetic regulator of gene expression, *DNMT3A* encodes a methyltransferase enzyme that catalyzes DNA methylation. The pathogenic mutations of this gene promote the self-renewal of HSC, consequently impacting all hematopoietic lineages, as well as promoting pro-inflammatory T-cell polarization and triggering the inflammasome complex [10].

*TET2* is the second most commonly mutated gene responsible for 20% of CHIP instances. By promoting the oxidation of DNA–methyl groups (demethylation), *TET2* works in contrast to *DNMT3A*. Additionally, it plays a role in transcription by recruiting histone modifiers [10,14]. *TET2* mutation has been linked to both in vitro and in vivo increased atherosclerosis and macrophage inflammatory gene expression [10,12].

*ASXL1*, with frequencies from 5–10%, is the third most mutated gene in CHIP and results in histone modification. The fact that *ASXL1* mutations are often present in myeloid malignancies is widely known. However, current research suggests these mutations also contribute to inflammation and mitochondrial impairment in T cells [10,15,16].

Additional genetic variations, such as *JAK2*, *TP53*, *PPM1D*, *SF3B1*, and *SRSF2*, are not as common and collectively account for less than 10% of the mutations observed in CHIP [10,11].

If CHIP fulfills all the diagnostic criteria mentioned in the previous chapter, except for the detected driver mutation with a minimum allele frequency of 2%, it would not be classified as CHIP in a strict sense [8,10]. However, clonal hematopoiesis without driver mutations still carries a substantial risk of mortality from any cause [17,18].

## 4. Clinical Manifestations of CHIP

CHIP poses a significant risk for the development of myeloid malignancies, such as AML, MDS and myeloproliferative neoplasms (MPN). Research indicates that cells that have individual mutations in the *DNMT3A*, *TET2* or *ASXL1* genes, the genes that are most commonly mutated in CHIP, are also frequently found in AML. Cells with these mutations, along with the leukemic cells that carry multiple mutations, may initiate the development of AML [19,20,21]. Genovese et al. showed that individuals displaying clonal hematopoiesis had a significantly higher probability of being diagnosed with hematologic cancer at least six months after DNA sampling compared to those without any observable suspected somatic mutations [18].

Research has suggested that the presence of CHIP increases the risk of death by 40%, which is notably greater than the impact attributed to blood cancers. This is supported by the findings of Jaiswal et al., where only one 1 of 246 individuals with this genetic mutation died from blood cancer [11,22]. They suggested that despite conventional CVD risk factors such as smoking, obesity, type 2 diabetes mellitus (T2DM), hypertension, hypercholesterolemia, coronary artery disease (HR 1.8–2.0), ischemic stroke (HR 2.6) and early heart attack (HR 4.0) were more likely to occur in patients with CHIP mutations [11,12,22].

Even after adjusting for potential confounding factors, Jaiswal et al. discovered that somatic mutations in genes known to cause hematologic malignancies were slightly but significantly related to an elevated risk of T2DM. Individuals diagnosed with T2DM had a slightly higher likelihood of possessing mutations compared to those who did not have T2DM. In addition, upon examining two groups consisting of 3353 individuals, they discovered a higher overall occurrence of coronary heart disease and ischemic stroke in individuals with a genetic mutation [22].

It is noteworthy that despite considering conventional risk factors, such as smoking, overall cholesterol levels and high-density lipoprotein cholesterol levels, the existence of a somatic mutation still showed a significant connection to the occurrence of coronary heart disease and ischemic stroke. Moreover, it was linked to a greater variant allele frequency (VAF) [22].

Another research conducted by Jaiswal et al. revealed that alterations in *DNMT3A, TET2* and *ASXL1* genes were linked to a 1.7-fold to 2.0-fold increased risk of developing coronary heart disease, whereas the *JAK2 V617F* mutation was linked to a 12-fold increased risk. It has also been demonstrated that CHIP is associated with a higher incidence of myocardial infarction (MI) in individuals under the age of 50. The two cohorts’ combined fixed-effects meta-analysis revealed a 4.0 odds ratio association between CHIP and early onset MI. CHIP was also associated with increased coronary artery calcification score as well as the risk of incident coronary heart disease. Both conditions showed a correlation with VAF [12].

Senguttuvan et al. conducted a comprehensive evaluation of publications regarding CHIP and CVDs, ultimately confirming a positive correlation between the two [23]. However, the correlation between somatic mutations and non-hematologic diseases may be caused by confounding factors, may be a common effect of the underlying aging process or may arise from intricate pathophysiological mechanisms with a particular emphasis on inflammation as a critical element in each phase of atherosclerosis [22,24].

## 5. Obesity

Obesity is currently one of the biggest health concerns on the rise. According to the World Health Organization (WHO), obesity prevalence has nearly tripled since 1975 [25]. It is defined as abnormal or excessive fat accumulation that may impair health. Obesity is associated with the development and progression of numerous pathological conditions, such as T2DM, dyslipidemia, hypertension and other CVDs [26,27,28].

A straightforward indicator for assessing total body fatness is the body mass index (BMI), which is determined by dividing the body weight in kilograms by the square of height in meters. The standard BMI range is stated as 18.5 to 24.9 kg/m^2^. Any individual whose BMI is ≥25 kg/m^2^ is regarded as overweight, and those with a BMI ≥ 30 kg/m^2^ are categorized as obese [25]. However, the BMI categorization standards established by WHO may not hold the same significance in some population groups since the percentage of body fat can vary considerably among individuals with the same BMI and is shown to be associated with different factors such as age, gender and race [29]. Therefore, regardless of BMI readings, it is critical to locate the distribution of fat, especially if it is an abdominal form that poses health risks due to abnormalities in hormone secretion and metabolic changes [27,30]. Th excessive accumulation of visceral fat, referring to fat storage in the intraperitoneal and retroperitoneal regions and a surplus deposition of ectopic fat, indicating fat stores in areas where fat is not naturally stored, such as the heart, liver, pancreas and skeletal muscle, are recognized as significant cardiovascular and metabolic risk factors, surpassing the implications of BMI [31,32]. These two entities typically occur in conjunction, as is evident in the case of the liver. Research has demonstrated that an overabundance of fat in the liver has been linked to an increase in cardiovascular risk comparable to those associated with visceral adipose tissue [32,33,34]. Furthermore, pericardial fat depots have been shown to correlate with the incidence of hard atherosclerotic CVDs, while epicardial adipose tissue was found to be strongly associated with anthropometric and clinical risk factors for CVDs [32,35,36]. The assessment of the waist circumference (WC) and the waist-to-hip ratio (WHR) as indicators of central adiposity are more effective measurements for identifying individuals at increased risk of developing atherosclerotic CVDs than BMI [37]. A WHR above 0.9 for men and above 0.85 for women suggests central obesity and is associated with the aforementioned risks [37,38]. Balkau et al. showed that CVD prevalence, adjusted for age, region and smoking status, correlated with WC in each BMI category [39]. Moreover, CVD was significantly associated with WC even among those with normal BMI [39]. Studies have also shown that measures of central obesity, such as WC and WHR, are directly and significantly correlated with an increased risk of mortality from all causes, thus highlighting the significance of promptly detecting individuals who are obese, utilizing a collaborative approach to treatment and ensuring the individuals and their caregivers are committed to achieving long-term goals, such as sustained weight loss [40,41,42].

For a long time, it was believed that the primary role of adipose tissue was to serve as a reservoir for the accumulation or depletion of surplus triglycerides. However, it has been established that it also functions as an endocrine gland that actively participates in numerous metabolic activities within the body [37]. This signifies that adipocytes produce and secrete biologically active substances, such as cytokines or complements [43]. Adipose tissue cytokines, referred to as adipokines, interact with the immune system, brain, heart, liver and muscles and play a role in the development of atherosclerosis and insulin resistance [37]. Crucial adipokines that play a role in previously mentioned processes are leptin, resistin, retinol binding protein 4, angiopoietin such as protein-2, IL-6 and MCP-1. These adipokines initiate widespread inflammation, the malfunction of the endothelial cells, excessive blood clotting and resistance to insulin, all of which contribute to the development of atherosclerosis [37,44,45]. Furthermore, the surplus deposition of adipose tissue in visceral and ectopic regions leads to hypervolemia. This, in turn, causes an increase in stroke volume, cardiac wall strain and myocardial damage, which can eventually culminate in heart failure [31]. Oxidative stress due to obesity-induced increases in saturated fatty acids, increased sympathetic nerve activation, as well as increased concentration of proinflammatory immune cells in the adipose tissue of obese individuals, also contribute to the development of CVD [37].

## 6. CHIP and Obesity—Current Evidence

To date, only a limited number of publications with the primary objective of investigating the link between CHIP and obesity has been published (Table 1).

Pasupuleti et al. obtained 47,466 unrelated participants who were free of T2DM at baseline and had adequate CHIP measurements from the UK biobank. The study sample consisted of 43.0% overweight and 23.6% obese individuals. A total of 5.8% of the participants were affected by CHIP, with the highest prevalence of mutations observed in the *DNMT3A* (3.7%) and *TET2* (1.0%) genes. The study reported that individuals with CHIP mutations had higher average WHR. Specifically, the existence of a CHIP mutation was linked to a 0.0028 rise in WHR, with a *p*-value of 0.03. Further analyses revealed that there was a mutual association between CHIP and WHR. CHIP prevalence increased with higher WHR: the proportion of subjects with CHIP was 4.93%, 5.75% and 6.56% in the first, second and third quartiles of WHR, respectively. This implies that dysfunctional metabolic activity could accelerate the growth of clonal hematopoiesis. Pasupuleti et al. further evaluated various mice models that possessed *TET2* ^−/−^*, DNMT3A ^+/−^, ASXL1 ^+/−^,* and *JAK2 ^+/−^* mutations, as well as the leptin-deficient *Lep ^Ob/Ob^ (Ob/Ob)* mutation. They demonstrated that both the compound mutant (*TET2 ^−/−^; Ob/Ob, DNMT3A ^+/−^; Ob/Ob, ASXL1 ^+/−^;Ob/Ob* and *JAK2 ^+/−^; Ob/Ob*) and CHIP mutant bone marrow (BM; *TET2 ^−/−^, DNMT3A ^+/−^, ASXL1 ^+/−^* and *JAK2 ^+/−^*) transplanted into *Ob/Ob* mice resulted in the rapid growth of mature myeloid cells and hematopoietic stem/progenitor cells, leading to a severe form of MPN/AML and CVD. This was linked with an increase in pro-inflammatory cytokines such as IL-1β, IL-6 and TNF-α, as well as intracellular Ca^2+^ levels [46].

The regulation of adipocyte lipid metabolism and triglyceride storage heavily relies on the presence of intracellular Ca^2+^. When the intracellular Ca^2+^ increases, it stimulates lipogenic gene expression, while simultaneously suppressing lipolysis, which eventually leads to adiposity [47]. Pasupuleti et al. assumed that higher levels of intracellular Ca^2+^ promote aberrant signaling, which could lead to an early onset of severe MPN/AML. To examine the impact of Ca^2+^ signaling inhibition on inducing clonal hematopoiesis in *Ob/Ob* specimens, they administered metformin (100 mg/kg, orally), nifedipine (100 mg/kg, orally), MCC950 (30 mg/kg, orally) and anakinra (10 mg/kg, i.p), either individually or in combination [46]. Although the evidence for nifedipine on lowering intracellular Ca^2+^ concentration is conflicting, Pasupuleti et al. demonstrated that the combination treatment significantly decreased the number of monocytes, neutrophils and white blood cells and increased red blood cell count, hematocrits and platelets count [46,48,49,50]. Moreover, the mentioned therapy reduced the frequency of leukemic blasts, undifferentiated stem cells, HSC and granulocyte macrophage progenitors, as well as diminishing the buildup of fatty deposits in the arteries of *Ob/Ob* recipients bearing CHIP through the inhibition of Ca^2+^ signaling [46].

Tercan et al. performed a study on 302 individuals who were overweight or obese (BMI > 27 kg/m^2^) from the 300 OB cohort. They detected 110 individuals who had mutations in clonal hematopoiesis driver genes, with 69 of them having *DNMT3A* mutations and 8 having *TET2* mutations [51]. The study reported that subjects with clonal hematopoiesis driver mutations and VAF > 2% had elevated levels of circulating IL-6 and IL-1β but no difference in CRP levels or peripheral blood mononuclear cells’ cytokine production capacity [51].

IL-6 and IL-1β are common cytokines that are elevated in CHIP, especially in the presence of *TET2* mutation [46,51,52,53]. The same cytokines are also increased in overweight and obese individuals [37]. For instance, IL-6 and the IL-6 receptors increase in correlation with the amount of visceral fat and positively correlate with both BMI and body fat percentage [37]. IL-6 has various metabolic functions, such as reducing the expression of glucose transporter-4 and insulin receptor substrate-1, which increases insulin resistance, as well as the decrease in lipoprotein lipase activity, leading to the uptake of lipids by macrophages and the formation of atherosclerotic foam cells and plaques. Additionally, IL-6 has pro-thrombotic effects on platelets [37,54,55,56]. Considering that CHIP, along with adiposity, increases the expression of the aforementioned cytokines, we can infer that CHIP and obesity synergistically contribute to the development of CVD and T2DM.

Moreover, the presence of *TET2* mutation in CHIP was found to be linked with increased insulin resistance in obese and elderly mice [57]. Tobias et al. showed that *TET2* and *ASXL1* mutations significantly correlated with a higher incidence of T2DM [58]. The expression of *DNMT3A*, the most frequently mutated gene in CHIP, was shown to be considerably elevated in macrophages derived from adipose tissue of mice subjected to a high-fat diet. Moreover, the excessive expression of *DNMT3A* strongly impeded the insulin-induced glucose absorption in adipocytes that were differentiated in vitro from stem cells derived from human adipose tissue [10,59]. The importance of *DNMT3A* in obesity pathophysiology was shown by Tovy et al., who analyzed adipose tissue in *DNMT3A* knockout mice. These mice’s adipose tissue showed abnormalities in the development of preadipocytes and the early activation of inflammatory genes, such as those involved in IL-6 signaling. The ultimate outcome was that those mice became excessively overweight because of adipocyte enlargement and tissue expansion [60]. Moreover, to investigate the impact of *DNMT3A* gene disturbance in myeloid cells, Sano et al. created a J774.1 myeloid cell line that lacks *DNMT3A* using the lentivirus/CRISPR technology. They showed that mice with inactivating mutations in *DNMT3A*, as well as *TET2*, exhibited more significant cardiac hypertrophy, diminished cardiac function and increased cardiac and renal fibrosis, along with elevated levels of pro-inflammatory cytokines [61].

Van Deuren et al. conducted a longitudinal study in which they followed 216 individuals with clonal hematopoiesis driver mutations and obesity over a period of 20 years. In 40 individuals, they observed that small clones could grow over time and become CHIP. The initial VAF ranged from 0.01% to 31.15% and increased on average by 7% annually. In addition, the rate of clonal expansion was positively correlated with insulin resistance and low levels of circulating high-density lipoprotein–cholesterol [62].

Haring et al. analyzed the association between lifestyle habits and CHIP. Their study consisted of 8709 postmenopausal women registered in the Women’s Health Initiative. These women had no prior history of cancer or CVD and had undergone comprehensive whole genome sequencing. At baseline, they evaluated four lifestyle factors that were included in the healthy lifestyle score: BMI, smoking status, physical activity and diet quality. The study sample had an 8.57% prevalence of CHIP. A higher healthy lifestyle score did not show any association with the presence of CHIP. Individual lifestyle factors, including diet quality, smoking habits or higher levels of physical activity demonstrated no association with CHIP. However, a normal or overweight BMI compared to BMI ≥ 30 kg/m^2^ was significantly associated with lower odds of CHIP [63]. The lack of evidence for an association between CHIP and other observed factors may be explained by certain methodological limitations, since unhealthy dietary habits, as well as low physical activity, are widely known risk factors for obesity, which was shown to be statistically significantly associated with CHIP [63]. Moreover, in the study of Dawoud et al., a strong association between *ASXL1* loss of function mutations and current smoking status was demonstrated [64].

On the other hand, Bhattacharya et al. recently reported significant effects of lifestyle modification on CHIP and CV outcomes in a cohort study of 44,111 adult participants from the UK Biobank [65]. The authors reported that the prevalence of CHIP consistently decreased as diet quality improved during 10-year follow-up. In fact, compared to the diet regarded as healthy, unhealthy diet was associated with 25% increased likelihood of the presence of CHIP development, without significant difference with respect to the distribution of CHIP-associated genes (*DNMT3A*, *TET2* and *ASXL1*). Moreover, the observed change in prevalence was accompanied by improved CV outcomes, as individuals eating unhealthily had more CV events when compared to individuals eating healthily, with the risk being even worse if patients had CHIP concomitantly.

Based on clinical evidence, CHIP carries a high risk of hematologic malignancy development and is associated with a poorer prognosis in people with solid tumors [16,17,18,19,20,21]. Similarly, obesity is also associated with an amplified risk of various types of tumors. Steele et al. noted that in 2014, about 631,000 individuals in the US were diagnosed with cancer related to being overweight or obese, accounting for 40% of all cancer diagnoses [66]. Harris et al. proposed several mechanisms through which obesity triggers and sustains carcinogenesis. They identified multiple local and systemic factors influenced by obesity, which could impact the cancer pathophysiological processes. Some of these are proinflammatory cytokines that are upregulated in obesity, including IL-1β, IL-6 and TNF-α. IL-6 is associated with tumorigenesis in terms of sustaining proliferative signaling, mutation and tumor-promoting inflammation, activating invasion and metastasis, genome instability and resisting cell death [67,68]. IL-1β is associated with invasion activation and metastasis, genome instability and mutation, tumor-promoting inflammation and immune destruction avoidance [67,69]. TNFα is linked with proliferative signaling sustenance, growth suppressors evasion, angiogenesis induction, invasion activation and metastasis, tumor-promoting inflammation and immune destruction avoidance [67,70]. Notably, these same cytokines are elevated not only in obese patients but also in patients with CHIP, indicating that these two conditions may act synergistically to promote cancer development [37,46,51,67].

Furthermore, there are indications that the occurrence of CVDs is associated with the emergence of cancer and vice versa. It is possible that these two entities may affect the advancement of one another [71]. Koelwyn et al. demonstrated that, in both mice and humans, MI promotes breast cancer development and cancer-specific mortality [72]. Kwak et al. showed that individuals who received percutaneous coronary intervention displayed an increased likelihood of developing cancer compared to the control group, particularly in relation to lung- and blood-related malignancies [73]. Despite being regarded as distinct illnesses, CVDs and cancer are acknowledged to have mutually shared risk factors, such as obesity, tobacco use, diabetes and advancing age [10]. One of the proposed shared pathophysiological mechanisms in cancer and CVDs development is inflammation. It is crucial for each phase of atherosclerosis and is also important in tumor growth and progression [24,71,74,75]. Considering that inflammation is a key feature of CHIP, as well as obesity, it can be inferred that the pathophysiology of all four conditions may be interconnected with inflammation [10,11,12,13,16,17,18,19,20,21,23,37].

**Table 1:** Studies examining the associations between obesity and CHIP.

Year	Study	Subject/Cell Characteristics	Results
2017	You et al. [59]	(1) *DNMT3A* knock-out mice on a high-fat diet (45% energy from fat)—in vitro and in vivo analysis (2) Human adipose tissue-derived stem cells	The overexpression of an *DNMT3A* was identified as a key epigenetic driver of insulin resistance in vitro and in vivo
2020	Fuster et al. [57]	Mice with *TET2*-deficient BM cells and fed with a high-fat/high-sucrose obesogenic diet (35.8% energy from fat and 36.8% from carbohydrate—primarily sucrose)	*TET2*-loss-of-function-driven clonal hematopoiesis exacerbated insulin resistance induced by high-fat/high-sucrose obesogenic diet
2021	Pasupuleti et al. [46]	(1) 47,466 participants, from which 2756 were identified with CHIP (2) Mice carrying CHIP mutations as well as leptin deficient mutation	- An increase in WHR was linked to the existence of the CHIP mutation. Additionally, CHIP prevalence rose in correlation with greater WHR - Obesity-driven CH was confirmed on mice models - A combination of metformin/nifedipine/MCC950/anakinra was demonstrated to be both secure and efficacious in averting CH and its associated risks
2021	Tercan et al. [51]	302 overweight or obese participants, of whom 110 were identified with clonal hematopoiesis driver mutations	Participants with clonal hematopoiesis driver mutations had higher circulating IL-6 and IL-1β levels
2021	Van Deuren et al. [62]	216 participants with clonal hematopoiesis driver mutations and obesity, followed over a period of 20 years	- Small clones have the potential to develop gradually and transform into CHIP - The rate of clone expansion was positively correlated with insulin resistance and low levels of circulating high-density lipoprotein–cholesterol
2021	Haring et al. [63]	8709 participants (postmenopausal women), of whom 746 were identified with CHIP	An individual who had a BMI within the normal or overweight range, in contrast to someone with a BMI of ≥30, exhibited a noteworthy correlation with decreased probability of CHIP
2021	Bhattacharya et al. [65]	44,111 participants followed over a period of 10 years, of whom 2507 were identified with CHIP	- Throughout the follow-up period, there was a consistent reduction in the incidence of CHIP as dietary quality improved - Unhealthy diet was linked to a 25% higher probability of CHIP development - Individuals who consumed an unhealthy diet had a higher incidence of CV events compared to those who maintained a healthy diet
2022	Tovy et al. [60]	(1) *DNMT3A* knock-out mice (2) The murine *3T3-L1* and *BAC-C4* preadipocyte cell lines	- Loss of *DNMT3A* prompted early activation of inflammatory genetic pathways, which led to a significant rise in stem cell pool and expanded adipocyte progenitors, which exhibited aberrant differentiation - *DNMT3A* had a comparable effect on preadipocytes as obesity induced by a high-fat diet

Abbreviations: CHIP—clonal hematopoiesis of indeterminate potential; BM—bone marrow; WHR—waist-to-hip ratio; CH—clonal hematopoiesis; BMI—body mass index; CV—cardiovascular.

## 7. Future Perspectives

**Figure 2:**
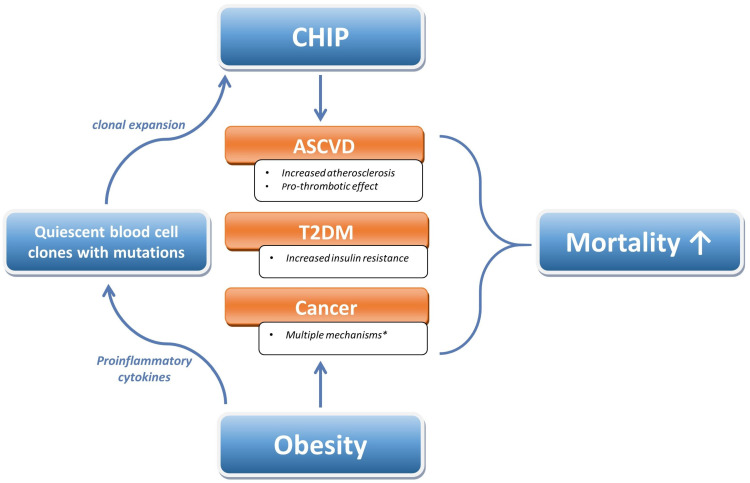
The vicious cycle of CHIP and obesity. Arrows represent the directions of the pathophysiological pathways. * Refer to the main text for further details. Abbreviations: CHIP—clonal hematopoiesis of indeterminate potential; CVD—cardiovascular disease; T2DM—type 2 diabetes mellitus.

Obesity has become a global pandemic, with at least 2.8 million people dying yearly due to being overweight or obese [25,76]. It is associated with a higher burden of cardiovascular diseases, diabetes, musculoskeletal disorders and certain cancers, as well as a higher mortality rate in comparison with persons with normal weight [25,26,27,28,37,66,67]. Although weight reduction strategies will yield more favorable outcome for most obese individuals, obese patients with CHIP may require even more attention for prevention and treatment measures due to their elevated risk of developing the aforementioned pathologies. Despite being a relatively novel concept, CHIP has been proven to contribute to the onset of CVD, T2DM and myeloid malignancies [12,19,20,21,22,23]. Although deep-targeted sequencing has indicated that most middle-aged humans have quiescent blood cell clones with different cancer-associated variations, the additional factors that alter fitness and contribute to the further clonal expansion of such cells to meet the criteria for CHIP are not yet understood. Poor dietary patterns and obesity have emerged as putative catalyzers of such development. However, so far, a scarcity of studies directly explored the correlations between obesity, CHIP and CHIP-associated pathologies [46,51,57,59,60,62,63,65]. Nevertheless, considering the available data, it can be inferred that obesity plays a universal role in actuating the development of CHIP. Furthermore, a conceivable vicious cycle seems to exist in which the pro-inflammatory state triggered by obesity and CHIP exacerbates the risk of progression for both ailments and amplifies the likelihood of developing CVD, T2DM and malignancies (Figure 2). Nonetheless, this inference necessitates further assessment through animal and in vitro studies, as well as observational and interventional studies with human participants. Further research is required to determine the precise extent of the increased susceptibility to developing CVD, T2DM, tumors and, ultimately, death in obese individuals with CHIP. This will enable us to make an informed decision as to whether it is necessary to actively screen for CHIP in the obese population, given the potentially significant rise in risk. Under those circumstances, CHIP should be deemed an unfavorable risk factor modifier, particularly in individuals with moderate cardiovascular risk, thereby placing them in a higher category that necessitates more rigorous and stringent treatment and monitoring. Regarding treatment choices for individuals who are overweight and have CHIP, only Pasupuleti et al. suggested focused therapeutic alternatives on animal models [46]. Nevertheless, these findings still bear no clinical utility, and it is therefore imperative to conduct additional research to create targeted therapy for overweight patients with CHIP in order to mitigate unfavorable outcomes with regard to CVD and obesity.

## 8. Conclusions

In conclusion, obesity and CHIP have been individually linked to a considerable risk of CVDs, malignancy and mortality through a number of studies. Nevertheless, the combined impact of these conditions is a novel idea that has yet to reveal the full extent of its clinical significance. Specifically, obesity seems to promote the clonal expansion of quiescent blood cell clones with cancer-associated variations. On the other hand, the pro-inflammatory phenotype associated with both CHIP and obesity triggers a cascade that subsequently increases the risk of the development of the above-noted pathologies.

## Data Availability

Not applicable.
